# When research seems like clinical care: a qualitative study of the communication of individual cancer genetic research results

**DOI:** 10.1186/1472-6939-9-4

**Published:** 2008-02-22

**Authors:** Fiona A Miller, Mita Giacomini, Catherine Ahern, Jason S Robert, Sonya de Laat

**Affiliations:** 1 Department of Health Policy, Management and Evaluation, University of Toronto, Toronto, Canada; 2 Department of Clinical Epidemiology and Biostatistics, McMaster University, Hamilton, Canada; 3 Department of Health, Aging and Society, McMaster University, Hamilton, Canada; 4 Department of Basic Medical Sciences, University of Arizona College of Medicine, Phoenix, USA

## Abstract

**Background:**

Research ethicists have recently declared a new ethical imperative: that researchers should communicate the results of research to participants. For some analysts, the obligation is restricted to the communication of the general findings or conclusions of the study. However, other analysts extend the obligation to the disclosure of individual research results, especially where these results are perceived to have clinical relevance. Several scholars have advanced cogent critiques of the putative obligation to disclose individual research results. They question whether ethical goals are served by disclosure or violated by non-disclosure, and whether the communication of research results respects ethically salient differences between research practices and clinical care. Empirical data on these questions are limited. Available evidence suggests, on the one hand, growing support for disclosure, and on the other, the potential for significant harm.

**Methods:**

This paper explores the implications of the disclosure of individual research results for the relationship between research and clinical care through analysis of research-based cancer genetic testing in Ontario, Canada in the late 1990s. We analyze a set of 30 interviews with key informants involved with research-based cancer genetic testing before the publicly funded clinical service became available in 2000.

**Results:**

We advance three insights: First, the communication of individual research results makes research practices *seem *like clinical services for our respondents. Second, while valuing the way in which research enables a form of clinical access, our respondents experience these quasi-clinical services as inadequate. Finally, our respondents recognize the ways in which their experience with these quasi-clinical services is influenced by research imperatives, but understand and interpret the significance and appropriateness of these influences in different ways.

**Conclusion:**

Our findings suggest that the hybrid state created through the disclosure of research results about individuals that are perceived to be clinically relevant may produce neither sufficiently adequate clinical care nor sufficiently ethical research practices. These findings raise questions about the extent to which research can, and *should*, be made to serve clinical purposes, and suggest the need for further deliberation regarding any ethical obligation to communicate individual research results.

## Background

In recent years, policy makers and scholars concerned with research ethics have declared a new ethical imperative: that researchers should communicate the results of research to participants [1,2]. For many commentators, the obligation is restricted to the communication of the general findings or conclusions of the study – aggregate information about the sample of participants [1]. But for others, the obligation is to disclose individual results, especially where these results are perceived to have clinical relevance. The obligation to disclose individual results is argued most forcefully in the case of genetic test results in genetic research (i.e., research-based genetic test results) [3-6], but increasingly the obligation is suggested for other types of clinical research, such as unblinding at the conclusion of trials and the reporting of relevant individual-level clinical data to participants [7,8]. Reasons for disclosure are many, with ethicists arguing that the principles of respect for persons, reciprocity, beneficience, and justice require the offer of research results to research participants. Yet several scholars have advanced cogent critiques questioning whether these principles are served by disclosure or violated by non-disclosure, and whether the communication of individual research results respects ethically salient differences between research practices and clinical care [9-13]. Empirical data on these questions are limited and available studies are inconclusive. On the one hand, studies regarding the preferences of research participants and the practices of researchers suggest a growing enthusiasm for the communication of aggregate and individual results to participants [1,2,14-17]. On the other hand, some research examining the experience of individual result disclosure highlights the potential for significant harm [18,19].

To date, advocates of the communication of research results have focused on practices and policy in research contexts. Yet *clinical *practices are also implicated by the communication of research results that are perceived to have clinical significance, especially research results about individuals. This paper explores the relationship between research and clinical care, and the implications of the disclosure of individual research results, through analysis of research-based cancer genetic testing in Ontario, Canada in the late 1990s. Drawing on interviews with participants, researchers, and other clinical providers, we gain three crucial insights. First, the communication of research results about individuals makes research practices *seem *like clinical services for participants (who identify themselves as 'patients'), researchers and other clinicians. Second, while valuing the way in which research enables a form of clinical access, our respondents experience these quasi-clinical services as partially inadequate. They express concerns about excessive delays in the receipt of research test results, the quality of research-based testing, and the ability of researchers to act with sufficient clinical sensitivity toward participants. Finally, our respondents recognize the ways in which their experience with these quasi-clinical services is influenced by research imperatives, but they understand and interpret the significance and appropriateness of these influences in different ways. In sum, our findings suggest that the hybrid state created through the disclosure of research results about individuals that are perceived to be clinically relevant may produce neither sufficiently adequate clinical care nor sufficiently ethical research practices. These findings raise questions about the extent to which research can, and *should*, be made to serve clinical purposes, and suggest the need for further deliberation regarding any ethical obligation to communicate individual research results.

### The case: research-based cancer genetic testing in Ontario, Canada

The genes associated with hereditary breast/ovarian and colorectal cancers were identified in the early and mid-1990s, with high expectations for their clinical utility. Early estimates of the lifetime risk associated with deleterious mutations in these genes were developed through study of limited numbers of very high risk families, and it was clear to informed commentators that additional epidemiologic research was needed to clarify risks in these and other populations [20]. In addition, it was not immediately apparent whether any interventions would be effective in reducing the risks that deleterious mutations in these genes conferred. Viewing research to answer these questions as both essential, and as a safer context in which to provide cancer genetic testing, relevant professional societies and other stakeholders advocated that cancer genetic testing only be conducted within the context of well designed research protocols [21,22]. Indeed, Frances Visco of the National Breast Cancer Coalition in the US argued in 1996 that, "Under existing circumstances, genetic testing for breast cancer outside of quality research protocols is harmful to your patients" [23]. This and other commentary tacitly presumed that genetic test research results would be disclosed to research participants.

In the US, the commercial imperative soon bypassed cautious guidance, with commercial genetic testing becoming available in 1996 [24]. In several other jurisdictions, however, research-based access to cancer genetic testing prevailed through the 1990s [25,26]. In Ontario, for example, access was limited to research protocols during this time, with several large epidemiologic research studies making it possible for individuals to receive research-based genetic test results regarding their risk for hereditary breast/ovarian and colorectal cancer from the mid-1990s on, at no cost to themselves. Two of these initiatives involved Ontario-wide, population-based registries: a familial breast and ovarian cancer registry, and a familial colorectal cancer registry [27,28]. A separate genetic epidemiologic research initiative, focusing exclusively on familial breast/ovarian cancer, was based in Toronto, and enrolled high-risk families [29,30]. It was not until April 2000 that full public funding for clinical cancer genetic testing services (breast/ovarian and colorectal) was provided to Ontarians through the province's health insurance program such that individuals could undergo cancer genetic testing, at no cost to themselves, outside the context of biomedical research [31].

## Methods

The data for this paper are drawn from a study of the evolution and organization of cancer genetics in Ontario, Canada, that received ethics review from the McMaster University Research Ethics Board. For the current project, we analyzed a set of 30 interviews with 30 key informants implicated in the delivery of research-based cancer genetic testing before the publicly funded clinical service became available (from a total of 83 interviews with 77 individuals for the project as a whole). Interviews were conducted in 2004 and 2005 with the written informed consent of the participant, and involved a retrospective discussion of the period under review. To support an analysis that is structured to address a bioethical debate concerning the roles and responsibilities of researchers and participants, we have classified our respondents into three categories: researchers (N = 22), research participants (N = 4) and clinicians (N = 4).

"Research participants" included in this analysis had received research-based genetic testing through one or several of the cancer genetic research studies discussed above. We found it hard to identify individuals who had been involved as research participants in the period under review. Our study was independent of the cancer genetic research discussed here so we could not make use of participant lists from those studies. Further, as this paper argues, the differences between research and clinical care were not always obvious to research participants or health care consumers: we know that several individuals we interviewed (but not those reported here) did not apparently pursue research-based testing, and we assume that many who did gain access through research protocols did not recognize themselves in our description of "research participants". The individuals we recruited were those who could be identified by relevant patient organizations or through Internet searches. To facilitate recruitment, and to minimize the risks of our research, our respondents played some public role: they were active members of patient support groups and some assumed the role of patient advocate. Because of these roles we asked these respondents to comment both on their personal experience with research-based access to genetic testing and on their perception of the experience of other research participants they counselled or worked with.

We classified individuals with varying degrees of responsibility for the research under review as "researchers." This includes scientists with no clinical role (e.g., laboratory geneticists) and clinicians (e.g., surgeons, oncologists) who were directly responsible for running relevant studies. This also includes clinicians (e.g., oncologists, clinical geneticists, genetic counsellors, clinical laboratory personnel) whose research involvement was more limited, including one or more of the following roles: (i) identification as investigator in a research grant proposal, (ii) membership on committees running relevant studies, (iii) employment as research staff (i.e., genetic counsellors employed to counsel research participants), (iv) involvement through recruitment, assessment or related activity with research participants, or supervision of clinical research staff.

Finally, we classified respondents (e.g., clinical geneticists) as "clinicians" where they did not assume responsibility for research-based cancer genetic testing yet had some connection to the relevant research studies (e.g., being co-located in centres where participants were recruited) – sufficient to allow them to comment meaningfully on what transpired.

The classification of research participants is straightforward, but the distinction drawn between clinicians and researchers (a category that includes clinicians) is more complex, reflecting the varied ways in which clinicians operate as researchers. Many of the clinicians here identified as researchers were clinicians first and foremost, but we have classified them as researchers for the purposes of this analysis because of their assumption of clear responsibilities in relevant research studies. Still, there are some few clinicians whose limited role in the relevant research renders their classification as researcher or clinician uncertain. In particular, all those we classified as clinicians in our sample had some engagement with the relevant research, largely by working in close proximity to research recruitment, and it is not always easy to decide whether the occasional clinical engagement with a research participant was subsequent to research participation, and thus a form of clinical follow-up, or concurrent as, for example, through the supervision of genetic counsellors who were employed (in whole or in part) as research staff. Further, some of those we classified as researchers played very similar day-to-day roles as those classified as clinicians, with a limited engagement with research participants and partial involvement in the management of relevant studies. In these few uncertain cases, we emphasized the degree of formal responsibility for research that each individual had assumed in making a final decision about classification.

We analyzed the data using a mixture of case study and modified grounded theory strategies [32-35]. As a case study, we were interested in understanding the phenomenon under study within its context, to analyze the data for its fit with theoretical propositions, and to search actively for contrary interpretations. In particular, we were interested in understanding whether the disclosure of research results might have clinical implications. From grounded theory we adopted the iterative and constant comparative analytic method [34], but with a more reflexive, hermeneutic approach to data interpretation [35]. Thus our coding strategy was mixed: we used a set of predetermined codes developed from our interview guide and our review of the literature to analyze the data, and also allowed codes to emerge empirically from (or "grounded" in) the data. We used qualitative data analysis software (NVivo, version 7) to assist in the organization and categorization of data. Interview transcripts were entered into our database and coded by members of the research team (FAM, CA and a research assistant). After reading each transcript or document in whole, to gain insight into the context and intent of the source, we then categorized the data into four broad pre-structured themes, drawn from our interview guide: the roles and beliefs of different health care providers; the connections between research and clinical service; resource allocation processes and rationales; and, the ways in which genetic service evolution in Ontario compared with other services or jurisdictions. For this paper, we focused our attention on data coded into the second theme about the transition from research practices into clinical care. While we asked respondents about this transition during the interviews, we did not systematically discuss the communication of research results; rather, this was an emergent theme.

As a final analytic step, we distributed copies of a draft manuscript to all cited respondents to ensure that we had not accidentally breached confidentiality and to solicit input on our interpretation of events. We received detailed feedback from two respondents who sought to clarify the operation and intention of the research studies, and the views of research participants. The final analysis presented here reflects the integration of these data and viewpoints.

## Results

Respondents discuss three main aspects of their involvement with research-based access to cancer genetic testing in Ontario. First, they clearly assume that genetic test research results must be communicated to individual participants as results become available during the course of the study. At the same time, respondents suggest that test result disclosure differentiates such studies from "pure" research, because it requires adjunct clinical services and entails clinical obligations among researchers, clinicians, and participants. Second, respondents express some dissatisfaction with the quality of research-based testing as a clinical service, and specify several ways in which such testing fails to satisfy clinical imperatives. Finally, respondents differ in their perceptions of how research imperatives influence the operation of research-based genetic testing. The informed consent process plays an important role in revealing the research-nature of these quasi-clinical practices. However, research priorities structure the terms and conditions of access to genetic test results in subtle ways not always apparent to respondents.

Participants and researchers share these views, but they sometimes emphasize different concerns, or describe these concerns in ways that reflect their different standpoints. Thus, in reviewing these three themes, we distinguish the parallel concerns of participants and researchers within each theme. Where relevant, we discuss the concerns of clinicians who were involved in associated clinical care alongside those of researchers.

### Theme 1: The communication of research results is expected, but disclosure makes research a quasi-clinical service

Respondents uniformly expect that research-based genetic test results that are perceived to be clinically relevant will be disclosed to individual participants. Yet these disclosures prove to be complex interventions that blur the line between research and clinical care, and create both clinical opportunities and clinical obligations.

Researchers feel duty bound to communicate the results of clinically relevant research test results to participants. Yet this type of disclosure is also seen as shifting the role of the study from "pure" research to clinical care.

How am I going to ask this person to my clinic and provide me with a blood sample and family history and just take that information and walk away and not give them back anything? (Researcher45)

...and I mean in a true research setting I think the argument would have been made that, you know, the results didn't necessarily go back to the participants in the study ... (Researcher42)

The research test results are disclosed because researchers perceive them to have clinical relevance. In turn, this clinical relevance creates clinical responsibilities for researchers.

...the patients that were recruited for the study were recruited for research. But from an ethical point of view we have to show that if we found anything that was of material interest to a patient that we had to have a system in place to provide service for that patient. (Researcher20)

In many other areas of research it can easily just be deemed as research and no immediate clinical impact, but this was different. This was the clinical information that we knew was useful, and data was emerging continuously. [pause]... and it affected patients. We had to deal with it. So even though it was research we had to do a clinical service. (Researcher44)

It is clear to researchers that the research test results will lead participants to make clinical decisions. In turn, these clinical decisions generate clinical demands on providers who are not directly involved in the research study.

The problem is that it is not pure research and it's not a pure service; it is a combination. It has always been from the beginning and that was one of our big problems ... This is definitely a service; women are taking action based on these test results; some of them are having their breasts removed, some of them are having their ovaries removed, you know, we can't view it as just research. (Clinician23)

I think we recognized that if, if a family participated in the research study and did qualify for testing and did, in fact, get results that we [clinical geneticists] would be the people picking up the pieces. We'd be giving the results and we'd be explaining the implications and we'd be answering their questions about, well, "What do I do with this information and how do I tell other members of my family?" Those would all be pieces that would essentially be clinical service. (Clinician3)

Participants expect to learn their research test results. Indeed, the individuals we interviewed participated in the research *in order *to get such information (although researchers informed us that not all participants wished to receive their genetic test results). Participants acknowledge that receiving research test results blurs distinctions between research and clinical care, but they dismiss such distinctions as unimportant given their desire for clinically relevant information.

... and so this was a blurred line because they had said that we would get a result from it but it was also a research protocol we were contributing to. So it was blurry. (Participant58)

And I realized that, you know, that there was this funny line that had been crossed and you know it was research and ah ... But when you're a patient in your, in the midst of all this, really you don't really give a damn. Your blood is sitting up there, you've been waiting for four months! You know? And it's like dangling a carrot in front of a horse. (Participant49)

### Theme 2. The research context limits the quality of the 'service' that is provided

The provision of research test results that are perceived to have clinical relevance changes the dynamics of research, creating clinical options for participants and clinical obligations both for the researchers and for clinicians outside the research study. Respondents express broad support for the clinical opportunities created, but they express concern about the quality of the clinical services provided under research auspices.

Researchers view service limitations as inherent in the use of research funds for the provision of clinical care, creating a classic quandary: the research cannot proceed without attending to clinical needs, but these needs cannot be fully addressed within the research context.

... questions raised about using research dollars to provide what some people perceived as being a diagnostic service and not really having sufficient funding to run it as a true service. So that caused some tensions at the consumer end of things. (Researcher42)

So it's one of those, it's a catch 22 situation, right? If you just do the research and offer no clinical feedback then people are upset about that, and if you do both and provide some clinical feedback people get upset that it's not a full-fledged clinical program, right? (Researcher45)

Researchers also express concern about the quality of research-based genetic testing. Complaints about the timely provision of genetic test research results are frequent, as is concern about the accuracy and reliability of these delayed results where produced in research, rather than clinical, laboratories. Further, researchers are unsure about the ability of some researchers to provide these effectively clinical services – to meet patient expectations for the sensitive provision of care.

A research lab [test quality] is less important. They have 100 samples, they care that 4% of the samples had mutations. They don't care that John Doe and Mrs. Jane Smith had a mutation. That's secondary to them. Whereas the clinical labs, that's what they're programmed to do, to deliver the right results to the right patient. (Researcher21)

[The researcher] was supervising the dissemination of results by mail. But I don't think [the researcher] had the best handle on how, although this was initially research ... and the patients didn't get results for years and years, eventually they did. I think it was set up, you know, as a molecular biology research project rather than something that was, in fact, going to become a provincial service. From the patient's end it was very much seen as a service. There was this dichotomy. (Researcher21)

For participants, the quality of clinical service provided through research protocols is also a concern, but different issues are emphasized. Like researchers, participants seem concerned about the limited access to research-based clinical services, and more specifically, the slowness of research test results and the poor bedside manner of some researchers acting in a quasi-clinical capacity. Unlike researchers, however, participants appear not to be aware of the risks to test quality created by the use of research laboratories.

I felt it should have been a service. No it wasn't. I didn't feel it was a service. I knew that it was research. It's just that as we waited and waited ... it was research with such clear application for us... (Participant58)

Probably, if you're involved in research, that is going to be your area of expertise, then that is what you're going to do best, and although you might try to give other services you're not going to be as good at it.... [The researcher] answered all the questions and all of that, it's just that when you're dealing with a genetic disorder or disease, or probability, it's very personal. It is as personal as it gets. And you do need that little bit of kid glove care, and that tender loving care, and you don't usually get that from researchers. (Participant56)

### Theme 3. Research provision of genetic test results remains research in morally salient ways

The research-based provision of genetic testing necessarily involves the provision of clinical care, however limited in quality. But even when research-based genetic testing addresses clinical needs it remains responsive to research imperatives. Research asks specific questions that may or may not match a patient's clinical questions, and answers them in accordance with its own timeframe and goals. Clinical services may be important, even essential elements of the research project, but the scope of these clinical services is determined by research priorities. For example, eligibility criteria for research-based genetic testing follow the parameters of a desired sample or study population, and not the requests of patients or their physicians. Participants do not generally anticipate such limits, which only come into focus when they encounter specific barriers or opportunities. Further, research participation imposes burdens, creating additional work for researchers and demanding time and energy from participants. In this context, the provision of test results serves an important research imperative: compensation and inducement for participation. The provision of research results offers a way of 'giving back' to participants for the contributions they make, and burdens they endure. It also serves as an important – even undue – inducement for research participation.

For researchers, the demands of research supersede those of clinical care. Where research questions suggest the need for different eligibility criteria than those required to meet clinical needs, the former are prioritized. Thus, research eligibility criteria sometimes rule out clinically needy cases. Conversely, research protocols sometimes make access easier for less clinically needy participants, as researchers pursue 'interesting cases' in lieu of needs-based clinical triage.

Respondent: Research works at its own pace, right? Because again, they have their vested interest in what they're looking for and what they are doing.

Interviewer: What do you mean?

Respondent: Well, they have a certain, they have a research project, whatever it may be, and somebody is providing the blood samples, yes? They're hoping that genetic testing is going to be completed at some point in time [....] so, depending on what their projects are, there might be priorities for who should get tested when. They might have an interest in, let's say, Jewish women with breast cancer, Jewish women with ovarian cancer, and those samples are going to get tested first, and everyone else is going to wait. So that's what I mean, like, there is a vested interest in it. They're looking at what interests them, and those samples are then going to be potentially processed sooner than others. So, whereas with a service test, it works a little bit differently. I mean, your sample goes in and it gets tested and there is a turn around time and you expect to get results in. (Researcher10)

For researchers, the need to apply complex eligibility criteria for access to research-based testing is one of the burdens of research participation. Together with the need for research data from participants (e.g., the completion of family history questionnaires, etc.), and the requirement for written informed consent, the application of eligibility criteria increases the workload for front line researchers, and is perceived to be burdensome for research participants, making additional demands on time and energy.

... and it [the existence of the clinical service] almost relieves us of the burden we have, because the research seemed to be kind of finicky. There was, you know, the eligibility criteria and then if this, then this, you know, you had to have a... I remember starting this. I had to have a flow chart; several flow charts. (Researcher33)

It's [clinical service access] less of a hassle for them [patients] because they're not part of a study and doing fifteen questionnaires before they get to a genetic counselling appointment. It's more like a, it's part of medical care rather than off at the side [....] I would imagine that it might seem a little more reassuring and a little more, trustworthy is not quite the right word, but because it's more of a part of the medical community rather than this research, we're going to send blood to Toronto, you've got to do some questionnaires. (Researcher7)

Researchers view access to research-based test results as one form of compensation to participants for the burdens of participation (others include, for example, clinical assessments of genetic risk). The potential receipt of research results is also an inducement for participation and is believed to improve participation rates.

Historically, I mean, historically up until, you know, the advent of Myriad and certainly before the establishment of the clinical program, we were kind of really the only game in Ontario – if you were a person who wanted a test for BRCA 1&2 [....] So, you know, we needed to have people participate, and then to have various people participating we would offer to share the results that we would get from our research with them, though we couldn't guarantee we could have their result in three months or six months, because that's the way research goes. (Researcher45)

... the patients did two questionnaires in the study and now it was sort of like, "Well, you do this for us and we'll do this for you." Like the testing and, you know, "We've done testing and you're going to also help us out with this study." (Researcher14)

Despite the offer of information as an inducement, and participants' motivation for information, researchers stress the importance of an explicit consent process to make clear that the research "contract" does not guarantee the participant timely access to research test results.

I think they tend to, I think, tended to deal with it quite well. It is also all in how you explain it. Somebody's giving a sample for research. There are no guarantees that a result is ever going to come out of it. And as long you explain it to people so that they understand what the situation is, they are giving informed consent. And then you would presume that they would realize that their results can take up to, you know, one or two years. (Researcher10)

Well the [research study] was trying to offer testing to the people who participated in that study but it was supposed to be made clear to those people that it was research testing that was being offered to them.... some people may have misunderstood that, you know [thinking] it was a test that they were, that they were going to definitely get, maybe [in] a certain time frame – versus a research test that could take a long time to develop, over time. (Researcher25)

The participants we spoke to indicate their own awareness of the differences between research and clinical care, but some express concern that others might not share this awareness, or care about its implications. Further, participants perceive the influence of research imperatives rather differently than researchers. Instead of highlighting the burdens of participation, or the role of research priorities in determining access to research-based genetic testing, participants point to more subtle ways in which they perceive their involvement in research to be valued, or not.

Participants seeking access to the clinical aspects of cancer genetic research projects are unsure that the research nature of such studies is consistently clear.

... see, that's where the confusion came for me and I was so surprised. I don't remember them identifying it as a research project to begin with. Never was it identified as a research project. Seriously. (Participant49) [NB: this respondent later identified an awareness of the research nature of the project]

But I think probably most, most people wouldn't make that distinction. They would think that they're getting the service and they wouldn't really think of themselves as being part of a research project, cause that's sort of going on in the background. You don't see that part of it. You kind of just see the service and maybe it's not always made that, you know, always made clear enough. I don't know. Or whether it would make a difference to some people that's also a question. It certainly wouldn't have made any difference to me, one way or the other. (Participant56)

The influence of research priorities on the terms and conditions of access to research based genetic testing is suggested by two participants. One views research as more flexible, improving her ability to gain access, while another sees research as restrictive, reducing her access to test results.

They were willing to take my blood on the basis that it was research and I think that, probably, they were able to be more flexible as a result. I don't know. That's how it came across to me. (Participant61)

... there were delays with the research protocols. We were caught in a research, so they couldn't prioritize, for instance, the blood of someone like me. (Participant58)

In addition to the direct influence of research priorities on access, some participants express a sense of being insufficiently valued through the research process – as if they were ignored, or treated as a means to an end.

You don't get information ... "We thought we would tell you what's going on in our research study ..." If we want to find it we have to look it up on the Internet. (Participant56)

...and then once you were in the research protocol it was like, you didn't exist as a patient anymore. They were obviously going to get back to you with a test result whenever ... (Participant58)

Finally, one participant wonders whether the inducement to participation created by the offer of research test results undermines truly voluntary participation.

So, you know, to only be able to get it through a research protocol, number one meant that, in a sense, you have to participate in the research. And I'm not anti-research .... But I think we have to be really careful with people when it's, when we're asking them to participate in something that benefits us, sort of professionally, and in other ways, you know? ... In essence, you're sort of forced into the research at times. This was a case in which there was no other way to get access to that test except to participate in research. So how could, what kind of choice is there really? (Participant58)

## Discussion

The technological capacity to identify mutations in the genes associated with hereditary cancer arose long before clarity existed about the epidemiological significance of these mutations in populations, or the clinical utility of obtaining advance information about these genetic risks in individuals. In Ontario and many other jurisdictions, research has been perceived to be the best way to manage this uncertainty as it allows a controlled and safe environment within which individuals might learn of their genetic risks and enables the collection of rigorous data to fill knowledge gaps. This sensibility, and historical pattern of practice, aligns well with emerging arguments in research ethics, which suggest that researchers have an ethical duty to disclose the individually-relevant results of genetic and other biomedical tests conducted under the auspices of research studies to research participants, especially where these results are perceived to have clinical significance. Yet the findings presented here give some cause for concern about a generalized ethical obligation to disclose individual research test results.

This study demonstrates that the disclosure of individual research test results can make cancer genetic research *seem *like clinical care, thereby creating unrealistic expectations as well as inadequate clinical service. Both researchers and participants recognize the distinction between research and clinical care, but the disclosure of individuals' test results blurs the line separating these domains. Research participants see themselves as "patients," first and foremost, and those who seek research test results do so with the clear intention to use these results to inform their own, or their families', cancer risk assessment and management (e.g., screening, prophylactic surgery, etc.). Researchers feel that test result disclosure alters the complexion of the research endeavor, making it something *other *than "pure" research. The entailed clinical obligations fall both on researchers and clinicians actively involved in the research projects, and on clinicians caring for the participants but not involved in the research.

Test result disclosure transforms research into a quasi-clinical service, but participants and researchers express concerns about the quality of the clinical service that can be provided under research auspices. The quality of – and access to – the service is inevitably compromised by the reliance upon limited research funds. While convinced of the need for these quasi-clinical services, respondents are dismayed by the amount of time it takes to get a test result, and researchers also express concern about potential quality problems with testing conducted in research laboratories. Further, participants and researchers express some concern about the ability of researchers to act with sufficient clinical sensitivity toward those seeking research test results.

Alongside quality limitations, the quasi-clinical services provided under research auspices are constrained by research imperatives that take priority over the objectives of individual clinical care. The views of participants and researchers largely agree here. Both participants and researchers are aware of an implicit contract in which research participation, and the burdens that it imposes, are exchanged for research test results. Yet the ways in which the demands of research structure the terms and conditions of access are not consistently visible to our respondents. Participants understand the distinction between research and clinical care, and know that they have been involved in research (though some wonder whether others would be so informed). But not all participants understand that their own access to research test results is conditioned by research priorities. Further, while participants do not discuss the general burdens of research participation – in contrast with researchers – they express concerns about how their participation is, or is not, valued over time. Finally, participants do not share the concerns of researchers regarding the potential for reduced accuracy and reliability of test results generated in research laboratories.

Much of the evidence gathered by other studies regarding the communication of research test results has revealed that participants generally want to receive either general research findings [15,17,36-38] or more individual research test results [14,16]. Indeed, because participants see themselves as contributing to research, they feel entitled to research test results in return [15]. While most researchers are willing to provide results to participants, many are concerned about the practical challenges of doing so [7,39,40]. Our study affirms the existence of participants' preferences to know, and researchers' general desire to meet these preferences. At the same time, a smaller body of work that investigates the meaning of result disclosure more qualitatively suggests that the communication of individual research test results is no simple add-on, but rather a complex, and potentially hazardous, intervention [18,19]. Paradoxically, our study affirms this finding as well.

Might the quasi-clinical services provided under research auspices ever be adequate? In Ontario, it is clear that cancer genetic test results provided through research protocols are often not up to prevailing clinical expectations of either timeliness or appropriateness of care. The degree of compromise may be specific to the case under study. In any event, proponents of the duty to disclose research test results have advanced a number of proposals that could improve service adequacy. In particular, steps might be taken to ensure the quality of the genetic test results that are disclosed to research participants [6], and sufficient clinical expertise could be made available to researchers so that participants can be treated with clinical sensitivity [5]. Further, research funders might provide sufficient monies to ensure that test result disclosure can be performed in a clinically adequate manner [41]. For example, additional funds might allow researchers to achieve a faster turn around time for research test results. These proposed remedies might solve several of the challenges of result disclosure identified in this case study.

However, not all of the concerns about the quality of the clinical services provided under research auspices are equally remediable. These quasi-clinical services will inevitably be driven by *research *priorities, and in some cases these will conflict with clinical imperatives. In particular, access to tests is determined by study sampling protocols, not by the priorities of clinical triage or fairness that should normally determine access to health services. For example, genetic epidemiological research may be organized to assess mutation prevalence in particular ethnic populations, making research unable to address the need for a test result in an individual outside that ethnic group. Individuals who seek testing may not be eligible to enroll in the necessary study, or once enrolled, may find themselves in a low priority group for timely results.

Even where clinical services in the research setting appear adequate, do the associated research practices remain ethically sound? A first concern involves the appropriateness of the reciprocity implicit in test result disclosure. As is apparent from our respondents' comments, and from literature on the duty to disclose, it is clear that the provision of research test results is seen as a form of recompense, whereby researchers "give back" to participants in recognition of their contributions to the research study. While many of our respondents view this reciprocity as appropriate, one participant feels it compromises the voluntariness of research participation. In the absence of parallel clinical access to these tests, this respondent views research participation as a requirement to gain access to testing [42]. Further, ethicists promoting the disclosure of research test results to participants see the communication of results as a way to demonstrate respect for research participants as full persons – to treat these persons as an end in themselves, rather than as means to an end [1]. Yet despite the promise of test result disclosure, some participants remain concerned about being insufficiently valued – even "used" by researchers as a means to an end. Test result disclosure is an unlikely remedy for participant alienation. While reciprocity is called for in research, it is not clear why individual test results might be the only, or best, gift to exchange.

An additional threat to research ethics in result disclosure arises from the therapeutic misconception [43,44]. This misconception occurs when participants fail to recognize the essential differences between the goals of research and the goals of clinical care, leading them to either, (1) believe that their own therapeutic needs will be met through a research protocol, or (2) ignore or fail to recognize the risks of research participation. Under the misconception of therapeutic value, participants provide inadequately informed consent. The therapeutic misconception can also affect researchers, leading them to mis-identify their role and to believe that their actions toward research participants are necessarily in the participants' clinical best interests[45].

Whether a therapeutic misconception exists where genetic research test results are disclosed depends largely on whether the risks entailed by the test information are downplayed. This study provides several reasons for believing that they may be. Participants seem unaware of researcher concerns about the quality of test results from research laboratories. They also seem inconsistently aware of how research priorities determine access, or timely access, to test results. Further, none of our respondents (participants or researchers) question the clinical significance of these investigational test results. This confident consensus is somewhat surprising. By the latter half of the 1990s, cancer genetic test results were known to identify clinically important risks. But the epidemiological research occurring in Ontario and elsewhere at this time was designed to clarify the risks arising from varied mutations in different populations, and to evaluate the clinical benefits of prophylactic interventions. Though not *without *clinical importance, we might expect respondents to express *some *uncertainty about the precise meaning and value of these investigational genetic test results.

The assessment of clinical importance is relevant to the threat of the therapeutic misconception: the clinical value of investigational test results might be overplayed and participants misled. But the challenges arising in the assessment of clinical significance extend beyond this specific threat to the nature of the adjudication process itself. What *does *determine clinical significance? Does the concurrent availability of commercial cancer genetic testing render research-based genetic test results "clinically significant"? And which authorities – patient organizations, coverage decision-makers, expert consensus groups, commercial providers, research teams – are empowered to make such a judgment? As we have argued elsewhere, the distinction that many ethicists have promoted in specifying the obligation to disclose research results – between definitive/validated and preliminary/unvalidated results [3-5] – is almost impossible to define and apply. It is one of the fundamental conceptual uncertainties complicating the putative obligation to disclose [46]. In the absence of clear adjudications or adjudication processes, judgments about the existence or degree of clinical importance are likely to be profoundly influenced by the contexts in which such judgments are made – with the possibility that judgments will be influenced by the clinical *feel *of research practices.

This study faces several limitations. We interviewed a small number of research participants and asked all respondents to recall practices during a time period approximately 5 years earlier. Our data do not permit us to fully explore considerable heterogeneity within the categories of "participant," "researcher," and "clinician," and important differences between specific research studies, and within research studies over time. We know that many "researchers" identify themselves as clinicians first and foremost, but we have categorized them as "researchers" because of their formal involvement with the studies under review. We also know that not all "participants" were involved in the same studies, or participated in the same way: some were offered research-based genetic testing when they sought an assessment of their risk status at a familial cancer clinic, while others deliberately sought access to research-based genetic testing. Still other participants (though none of those interviewed by us) would have been contacted by a provincial cancer registry and offered research-based genetic testing, either because of familial risk or to serve as healthy "controls". Further, as individuals with public roles, the research participants we interviewed are not typical and their reflections on what other participants might experience or believe must be approached with caution.

However, these limitations do not detract from our conclusion that further consideration should be given regarding any ethical obligation to offer individual research results to research participants. As it has been advanced to date, this putative ethical obligation faces surprisingly few conditions. Most guidance makes clear that researchers should offer individual test results on a voluntary basis [1,5]. While this voluntarism is necessary, it may not be sufficient. Voluntarism cannot, after all, ensure that the offer of results does not serve as undue inducement to participate in research in the first instance. Further, as Parker notes, the offer of disclosure carries with it the "suggestion that one would be imprudent to refuse" [10]. Finally, while some commentators argue that all individual research test results should be offered to participants [8,47], most argue that results must meet some standard of reliability or significance [3-5]. Yet in the absence of clear and objective standards of clinical significance – standards that are often unavailable – the requirement of "clinical significance" as the trigger of an ethical obligation to disclose may prove idiosyncratic and thus morally suspect – responding to the contexts in which such judgments are made rather than the substantive elements of clinical validity and utility.

Beyond the constraints of voluntarism and test result significance it is assumed that disclosure is ethically meritorious. But what is required to gain these benefits, and can they invariably be achieved? Must research policy be altered to ensure that research projects that involve individual result disclosure also ensure that this clinical information is given in the context of a high quality clinical service? If not, do the partial and potentially limited clinical services that *can *be provided in typical research settings exacerbate participant alienation, and cause possible harm? The issues extend beyond the adequacy of the clinical services provided under research auspices to the ethics of the research itself. Our findings suggest that the clinical sensibility that prevails where clinically relevant research test results are disclosed may in fact create therapeutic misconceptions. Researchers may find it difficult to act ethically in seeking informed participation when result disclosure is seen as the reciprocal gift. And persons who participate *in order to get *individual research results may never provide fully informed consent. Finally, research may prove unable to fulfill its evaluative function where it is so pervasively viewed as the source of a clinically useful commodity.

## Conclusion

It is not our intention to cast doubt on the excellence of the goals or operation of the cancer genetic research studies considered here. These studies established much of the clinical expertise and institutional capacity for the clinical services that were subsequently developed in Ontario. They contributed essential knowledge about the epidemiology of mutations predisposing to cancer, and they significantly advanced knowledge about appropriate ways to manage inherited cancer risks. It is precisely because these studies were both well conducted and necessary that we need ask whether they (and others like them) should *also *serve clinical goals.

Further, in calling into question the obligation to communicate individual research results to research participants we are not advocating a return to a more paternalistic era when experts withheld vital information. Nor do we challenge ethical obligations to respect and value the contributions of research participants. Rather, we question whether individual research results are the appropriate token of that valuation, and whether the up-front "contract" with research participants should *always *involve the offer of individual research results. In our view, the routine disclosure of individual research results that are perceived to be clinically relevant blurs an ethically important distinction between research and clinical care [9-13]. Disclosure could deceive or unduly induce the involvement of research participants; it could also hasten the adoption of clinically unproven health technologies. Paradoxically, institutionalized testing through research protocols may *delay *the introduction of clinical services for reasons other than lack of evidence – as has been suggested to us with respect to the cancer genetic research studies in Ontario.

As we have argued elsewhere [46], there is a clear need for more debate and conceptual development regarding any obligation to disclose individual research results to research participants. Disclosure *can *be ethically appropriate, even essential, under the right circumstances. But more evidence and conceptual development is needed to consider both the full range of implications suggested here, and to identify morally salient differences in the research and clinical contexts through which researchers provide, and participants seek or gain access to, individual research results.

## Competing interests

The author(s) declare that they have no competing interests.

## Authors' contributions

FAM led the study, developed initial interpretations of the data and drafted and revised the manuscript. MG reviewed initial data analysis memos and made substantial revisions to draft versions of the manuscript. CA conducted the interviews, assisted with the data analysis and reviewed versions of the manuscript. JSR and SdL reviewed initial data analysis memos and suggested revisions to versions of the manuscript. All authors read and approved the final manuscript.

## Pre-publication history

The pre-publication history for this paper can be accessed here:


